# Impact of national COVID-19 restrictions on incidence of notifiable communicable diseases in England: an interrupted time series analysis

**DOI:** 10.1186/s12889-022-14796-0

**Published:** 2022-12-12

**Authors:** Katrina Nash, Jo Lai, Karanbir Sandhu, Joht Singh Chandan, Saran Shantikumar, Fatai Ogunlayi, Paul C. Coleman

**Affiliations:** 1 Oxford University Clinical Academic Graduate School, Oxford, OX3 9DU UK; 2 Reading, Royal Berkshire Hospital, London, RG1 5AN UK; 3 College of Medical and Dental Sciences, University of Birmingham, Birmingham, B15 2TT UK; 4 Institute of Applied Health Research, University of Birmingham, Birmingham, B15 2TT UK; 5 Warwick Medical School, University of Warwick, Coventry, CV4 7AL UK; 6 Centre for Food Policy, City University of London, London, EC1V OHB UK

**Keywords:** COVID-19 restrictions, Non-pharmaceutical interventions, Communicable diseases, Interrupted time series analysis

## Abstract

**Background:**

Non-pharmaceutical interventions (NPIs), such as travel restrictions, social distancing and isolation policies, aimed at controlling the spread of COVID-19 may have reduced transmission of other endemic communicable diseases, such as measles, mumps and meningitis in England.

**Methods:**

An interrupted time series analysis was conducted to examine whether NPIs was associated with trends in endemic communicable diseases, using weekly reported cases of seven notifiable communicable diseases (food poisoning, measles, meningitis, mumps, scarlet fever and pertussis) between 02/01/2017 to 02/01/2021 for England.

**Results:**

Following the introduction of COVID-19 restrictions, there was an 81.1% (95% CI; 77.2–84.4) adjusted percentage reduction in the total number of notifiable diseases recorded per week in England. The greatest decrease was observed for measles, with a 90.5% percentage reduction (95% CI; 86.8–93.1) from 42 to 5 cases per week. The smallest decrease was observed for food poisoning, with a 56.4% (95%CI; 42.5–54.2) decrease from 191 to 83 cases per week.

**Conclusions:**

A total reduction in the incidence of endemic notifiable diseases was observed in England following the implementation of public health measures aimed at reducing transmission of SARS-COV-2 on March 23, 2020. The greatest reductions were observed in diseases most frequently observed during childhood that are transmitted via close human-to-human contact, such as measles and pertussis. A less substantive reduction was observed in reported cases of food poisoning, likely due to dining services (i.e., home deliveries and takeaways) remaining open and providing a potential route of transmission. This study provides further evidence of the effectiveness of non-pharmaceutical public health interventions in reducing the transmission of both respiratory and food-borne communicable diseases.

## Background

The World Health Organisation declared severe acute respiratory syndrome coronavirus 2 (SARS-CoV-2) a public health emergency on 31st January 2020. Within one year, SARS-CoV-2 had infected more than 100 million people globally, resulting in over 2 million deaths [1]. In response to the public health emergency, countries globally implemented a range of pharmaceutical (e.g., vaccines) and non-pharmaceutical interventions (NPIs) (e.g., national lockdowns, social distancing, isolation policies, contact tracing systems and travel restrictions) to reduce rates of COVID-19 infection [2, 3].

While pharmaceutical interventions are disease specific, NPIs have been shown to suppress transmission of a broad spectrum of endemic notifiable infectious pathogens, such as seasonal influenza [4–6] and measles [7], in countries including, but not limited to, Australia, China, Germany and the United States of America [8–11]. The impact of NPIs on disease spread via respiratory droplets is supported by Bruggemann et al.’s [12] analysis of surveillance data from 26 countries, which demonstrated significant reductions in transmission of bacterial infections from *Streptococcus pneumoniae*, *Haemophilus influenzae*, and *Neisseria meningitidis* following the introduction of NPIs.

Public health measures to suppress the spread of COVID-19 were first introduced in England on March 23, 2020 [13], with members of the public instructed to stay at home and all non-essential businesses closed. As observed in other countries, it is likely that NPIs implemented in England to suppress transmission of SARS-CoV-2 would have reduced transmission of other endemic notifiable diseases.

To our knowledge, no study to date has utilised national data from Public Health England (now known as UK Health Security Agency following organisational restructure in 2021) to assess the impact of lockdown measures on transmission of communicable diseases in England. This study, therefore, aimed to identify whether the introduction of measures designed to reduce COVID-19 transmission affected the incidence of notifiable communicable diseases in England (Table 1).

**Table 1:** Infectious notifiable diseases and their route of transmission

Notifiable infectious disease	Description	Route of transmission
Food poisoning (campylobacter, salmonella, listeria, Cyclospora and shiga-toxin producing E.coli combined)	Public Health England defines food poisoning as ‘an illness caused by the consumption of food contaminated with bacteria, parasite, virus, chemical or other toxin	Faecal-oral
Measles	Measles morbillivirus (virus)	Respiratory
Meningitis (meningococcal meningitis)	Meningococcal meningitis caused by Neisseria meningitidis (bacterium)	Respiratory
Mumps	Mumps virus	Saliva or respiratory
Scarlet Fever	Streptococcus pyogenes, or group A streptococcus (GAS) (bacterium) that also cause impetigo	Saliva or respiratory
Pertussis (whooping cough)	Bordetella pertussis (bacterium)	Saliva or respiratory

## Methods

This study examines the impact of NPIs, specifically the introduction of a national ‘lockdown’ (stay at home orders and closure of schools and non-essential business), on cases of notifiable diseases in England. An interrupted time series analysis (ITSA) was considered an appropriate statistical method for evaluating the longitudinal effect of NPIs on reported cases of notifiable diseases, as it enabled comparison before and after intervention.

### Outcome and intervention

Due to pre-pandemic widespread prevalence, the outcome of interest for the study was the case frequency per week of: measles, mumps, meningococcal meningitis, pertussis, scarlet fever, and food poisoning (Table 1). The intervention was defined as introduction of a national lockdown, which occurred nationally on March 23, 2020 in England.

### Data sources

The data source for the study was weekly reported cases of diseases of interest from Public Health England’s notifications of infectious diseases (NOIDs) dataset for England [14].

### Statistical analysis

The ITSAs were conducted using segmented regression with a quasi-Poisson model with weekly cases of notifiable disease as the dependent variable. As the outcome was count of cases, Poisson regression model was considered most appropriate – other regression models such as ordinary least squares (linear) regression for continuous outcomes was considered inappropriate. An assumption of a Poisson distribution is that the variance is equal to the expected count. Initial analyses suggested variance was greater leading to overdispersion of the data, so a quasi-Poisson model was used. A second assumption was that observations are independent and autocorrelation was assessed by examining the plot of residuals and the partial autocorrelation function. It was hypothesised a priori that the intervention would result in a level (step) change in the outcome, given the restrictions that were associated with the NPIs.

The ITSA model included week as a linear variable to model for an underlying linear time trend and NPIs (national ‘lockdown’) as intervention dummy variables, coded ‘0’ for the pre-intervention period and ‘1’ for the post-intervention period. The break point for the model was March 23, 2020 signifying the introduction of national restrictions. The pre-intervention time period therefore was from week of January 02, 2017 to week of March 16, 2020 – and the post-intervention time period was from week of March 23, 2020 to January 02, 2021 for this analysis.

The model was further adjusted for seasonality in the underlying reported cases, using a harmonic term based on the week of the year and using two sine/cosine pairs per 12-month period. We have not considered inclusion of a recovery slope as the focus of the study was on the effect of national restrictions on reported cases of notifiable diseases in the immediate period after the lockdown, and no sensitivity analyses were conducted.

All data were analysed, and all plots generated, using RStudio (version 1.4.1103).

## Results

**Fig. 1:**
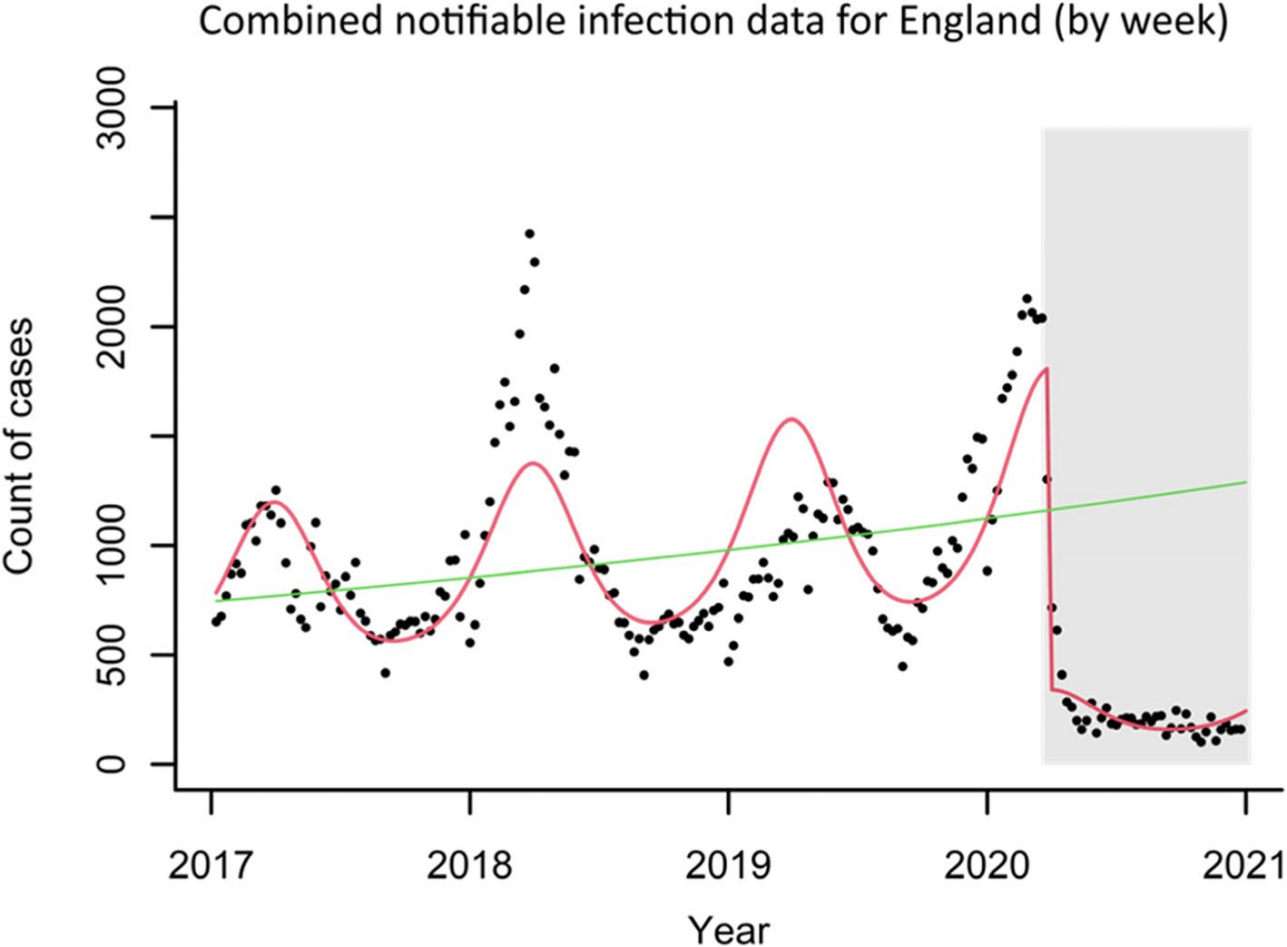
Incidence of notifiable infectious diseases with models adjusted for linear time trend and seasonality. England combined data for food poisoning, measles, meningitis, mumps, scarlet fever and pertussis. (Red line shows the trend based on the seasonally adjusted regression model; green line shows the de-seasonalised trend; grey box represents the time post-lockdown.)

Following the introduction of NPIs in England on March 23, 2020 there was an 81.1% (95% CI; 77.2–84.4) adjusted percentage reduction in the total number of notifiable diseases recorded per week, from an average of 990 cases per week before the introduction of national restrictions to 217 cases per week following the introduction of national restrictions (Table 2 and Fig. 1).

**Table 2:** Weekly incidence of notifiable infectious diseases in England before and after introduction of non-pharmaceutical interventions on March 23, 2020 [14]

Notifiable infectious disease	Mean weekly cases before introduction of non-pharmaceutical interventions	Mean weekly cases after introduction of non-pharmaceutical interventions	Unadjusted percentage reduction	Adjusted^a^ percentage reduction (95% confidence intervals)
England-Combined	990.4	217.3	78.1%	81.1% (77.2%—84.4%)
England -Food Poisoning	191.1	83.4	56.4%	48.6% (42.5%—54.1%)
England -Measles	42.3	5.3	87.5%	90.5% (86.8%—93.1%)
England -Meningitis	7.0	0.8	88.6%	82.8% (72.9%—89.1%)
England -Mumps	250.6	82.2	67.2%	88.2% (84.5%—91.0%)
England -Scarlet Fever	434.8	37.3	91.4%	88.6% (81.6%—93.0%)
England -Pertussis	64.6	8.5	86.8%	90.1% (86.9%—92.5%)

^a^Adjusted for linear time trend and seasonality

The greatest decrease in England was observed in weekly cases of measles, with a 90.5% percentage reduction (95% CI; 86.8–93.1) from 42 to 5 cases per week, followed by cases of pertussis (90.1% reduction), scarlet fever (88.6% reduction), mumps (88.2% reduction) and meningitis (82.8%). The reduction in the total number of food poisoning incidents was 56.4% (95%CI; 42.5–54.2), from 191 cases per week to 83 cases.

## Discussion

A total reduction in the incidence of endemic notifiable diseases was observed in England following the implementation of NPIs aimed at reducing transmission of SARS-COV-2 on March 23, 2020. The greatest reductions were observed in diseases most frequently observed during childhood that are transmitted via close human-to-human contact, such as measles and pertussis. These diseases follow similar routes of transmission as observed for SARS-COV-2 [15]. A less substantive reduction was observed in reported cases of food poisoning, defined by Public Health England as an illness caused by the consumption of food contaminated with bacteria, parasite, virus, chemical or other toxin.

Within England, around one-third of measles cases are international travel related [16], with secondary infection occurring within nursery, school and other educational settings. Travel restrictions [17] and school and nursery closures likely halted initial incursion and subsequent onward transmission of measles, as observed in countries across the European Union [7], including reduced incidence of measles among children in Germany [10]. In addition to measles, COVID-19 travel restrictions and closure of educational settings likely reduced transmission of other vaccine-preventable childhood infections, including mumps and pertussis. This is in line with research predating COVID-19 which highlights the role of educational settings in facilitating transmission of childhood respiratory infections [18, 19], as well as research conducted in Finland during the COVID-19 pandemic showing a decrease in daily emergency department visits for viral respiratory tract infections following nation-wide school closures [20].

In contrast to pathogens transmitted from person-to-person via saliva or respiratory droplets, cases of food-borne pathogens – typically transmitted via the faecal-oral route – displayed a less severe decrease. While enhanced levels of hand-hygiene, closures of educational settings and orders to work from home will have reduced human-to-human transmission of gastro-intestinal infections, many dining services (i.e., home deliveries and takeaways) remained open, providing a potential route of transmission. Findings are consistent with research from Germany and the USA which found a less severe reduction in food-borne pathogens when compared to respiratory infections following introduction of NPIs [7, 10].

Evidence presented here and from other global studies [8–11, 21, 22] suggest that NPIs to suppress the transmission of SARS-CoV-2, such as international travel restrictions, closures of schools and orders to work from home, led to reduced incidence of respiratory and food-borne pathogens. However, widespread disruption of public health and front-line health services are likely to have also resulted in reduced reporting and recording of notifiable infectious diseases, as observed via a decrease in routine reporting of sexually transmitted diseases within the USA during the pandemic [23].

Our study indicates that NPIs are an effective measure to reduce transmission of communicable diseases, in particular those transmitted via close human to human contact. However, this is balanced against the health, economic and social impacts of COVID-19 restrictions [24]. Findings presented here indicate that the incidence of notifiable diseases, such as measles, may be effective as a proxy to evaluate the effectiveness of restrictions on transmission of COVID-19, particularly in countries with inadequate COVID-19 testing and reporting facilities.

Study limitations include the omission of data on incidence of influenza due to insufficient data availability [14]. There may also have been significant underreporting during the COVID-19 pandemic, as patients may have been less likely to present to hospital, there may have been delays in reporting and there may have been diagnostic uncertainty surrounding the new disease (e.g., scarlet fever has similar symptoms to COVID-19). Furthermore, there were discrepancies between the sum of weekly reported cases reported for each year and the annual total reported. Nevertheless, we found this to be non-random which would reduce the risk of bias. A key assumption made was that without the NPIs (national ‘lockdown’), the pre-intervention trend in reported cases of notifiable infectious diseases would continue unchanged into the post-intervention period. It was not possible to add control group to our study as the intervention was a national lockdown. Finally, as the focus of this study was to determine whether or not the national lockdown in England had an impact on infectious disease notification frequency, and not to develop a prediction model, we did not undertake cross validation. Indeed, the dataset used only had one value for notifications nationally for each week and disease, so it was not possible to create training and validation sets.

In summary, this study provides further evidence of the effectiveness of NPIs in reducing the transmission of both respiratory and food-borne communicable diseases. These findings could be used to inform scientific modelling and decisions regarding NPIs when faced with future outbreaks of disease.

## Data Availability

The data sets analysed in this study are publicly available in the UK government Notifications of infectious diseases, available at https://www.gov.uk/government/collections/notifications-of-infectious-diseases-noids.
